# An Overview of Treatment Approaches for Octahydro-1, 3, 5, 7-tetranitro-1, 3, 5, 7-tetrazocine (HMX) Explosive in Soil, Groundwater, and Wastewater

**DOI:** 10.3390/ijerph192315948

**Published:** 2022-11-30

**Authors:** Varsha Srivastava, Grzegorz Boczkaj, Ulla Lassi

**Affiliations:** 1 Research Unit of Sustainable Chemistry, Faculty of Technology, University of Oulu, FI-90014 Oulu, Finland; 2 Department of Sanitary Engineering, Faculty of Civil and Environmental Engineering, Gdansk University of Technology, G. Narutowicza St. 11/12, 80-233 Gdansk, Poland; 3 EkoTech Center, Gdansk University of Technology, G. Narutowicza St. 11/12, 80-233 Gdansk, Poland

**Keywords:** AOPs, bioremediation, explosives, Fenton process, phytoremediation, wastewater treatment

## Abstract

Octahydro-1, 3, 5, 7-tetranitro-1, 3, 5, 7-tetrazocine (HMX) is extensively exploited in the manufacturing of explosives; therefore, a significant level of HMX contamination can be encountered near explosive production plants. For instance, up to 12 ppm HMX concentrations have been observed in the wastewater effluent of a munitions manufacturing facility, while up to 45,000 mg/kg of HMX has been found in a soil sample taken from a location close to a high-explosive production site. Owing to their immense demand for a variety of applications, the large-scale production of explosives has culminated in severe environmental issues. Soil and water contaminated with HMX can pose a detrimental impact on flora and fauna and hence, remediation of HMX is paramount. There is a rising demand to establish a sustainable technology for HMX abatement. Physiochemical and bioremediation approaches have been employed to treat HMX in the soil, groundwater, and wastewater. It has been revealed that treatment methods such as photo-peroxidation and photo-Fenton oxidation can eliminate approximately 98% of HMX from wastewater. Fenton’s reagents were found to be very effective at mineralizing HMX. In the photocatalytic degradation of HMX, approximately 59% TOC removal was achieved by using a TiO_2_ photocatalyst, and a dextrose co-substrate was used in a bioremediation approach to accomplish 98.5% HMX degradation under anaerobic conditions. However, each technology has some pros and cons which need to be taken into consideration when choosing an HMX remediation approach. In this review, various physiochemical and bioremediation approaches are considered and the mechanism of HMX degradation is discussed. Further, the advantages and disadvantages of the technologies are also discussed along with the challenges of HMX treatment technologies, thus giving an overview of the HMX remediation strategies.

## 1. Introduction

The application of diverse kinds of explosives in propelling rockets, the military, artilleries manufacturing, the mining industry, and construction is well recognized [1,2,3]. One of the most commonly used explosives is octahydro-1, 3, 5, 7-tetranitro-1, 3, 5, 7-tetrazocine, also referred to as high melting explosive (HMX). HMX is also widely recognized as an octogen as well as cyclotetramethylenetetranitramine [4]. Some important applications of HMX include the manufacturing of explosives and nuclear devices, utilization as raw material in propellant formulations, and artillery shells as burster chargers [5,6,7,8]. Additionally, HMX is often used as a vital component of secondary explosives, viz., HTA-3 [8]. 

**Figure 1:**
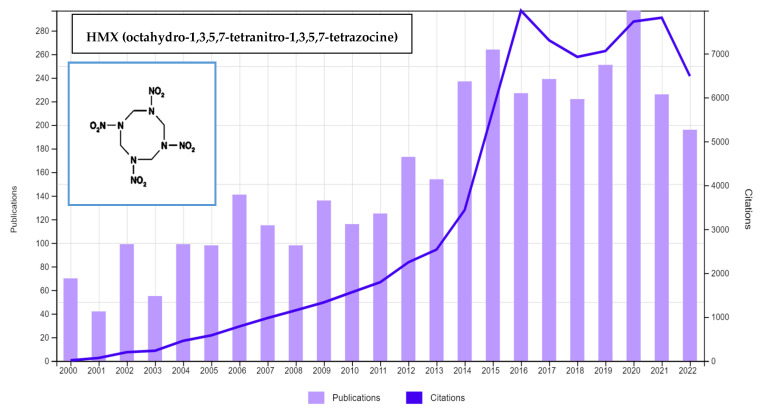
The trend of HMX-related investigations from 2000 to 2022 (based on https://www.webofscience.com/ accessed on 22 November 2022, keywords-HMX).

Owing to its high stability, high detonation heat, and high detonation velocity, HMX has been the most extensively exploited energetic chemical for both military and commercial applications [5,9,10]. HMX is a heterocyclic compound consisting of an eight-membered ring [11,12]. HMX can exist in four different crystalline phases, viz., α, β, δ, and γ [13,14]. Under ambient conditions, β-HMX is recognized to be thermodynamically stable among all phases and δ-HMX is usually more vulnerable than β-HMX. The bandgap influences the sensitivity of the four HMX polymorphs. The order of sensitivity for HMX polymorphs is β-HMX < γ- HMX < α- HMX < δ- HMX [13]. Figure 1 depicts the trend in HMX-related publications and citations from 2000 to 2022, demonstrating the significance of HMX-based investigations.

Explosive materials and/or their residues can be detected around explosive production plants, military firing ranges, or munitions utilization areas [12,15,16]. A significant amount of explosive-contaminated wastewater is released during the production of explosives which contaminates the surface water resources [6]. It is noteworthy that because of their high mobility in the soil as well as low adsorption, HMX can also cause groundwater contamination [8,17,18]. Additionally, the existence of HMX in the soil can adversely affect the soil profile and the diversity of microbial communities can be reduced which affects the natural degradation capabilities of soil with explosive contaminants. Various groundwater, wastewater, and soil samples have been reported to contain varying amounts of HMX (Table 1).

The occurrence of explosive pollutants in the aquatic environment and soil causes severe environmental pollution [26]. Contamination caused by the release of large amounts of HMX has become a compelling environmental dilemma across the world [26,27]. A low octanol-water partition coefficient (k_ow_ 0.06) [28], as well as poor water solubility-5 mg/L at 25 °C [29], makes HMX a recalcitrant chemical, and its long-term persistence in the environment warrants its sustainable removal [6,8]. Soil and water contamination by different explosives are well known but there has been no substantial progress on treatment approaches and understanding of the fate of explosives and their byproducts [7].

HMX is known to be mutagenic and carcinogenic for human beings and is classified as a Class D carcinogen [17,27,30]. Exposure to HMX can trigger acute poisoning, epilepsy, nausea, convulsions, and loss of consciousness [31]. HMX accumulation in the heart, kidney, liver, and brain has been reported and additionally, the liver and central nervous system can also be affected by HMX [31]. Furthermore, the degradation byproducts (as a result of the environmental impact) of HMX can also pose severe environmental threats even if present in low concentrations [32]. The detrimental impact of HMX on the soil profile is well documented as it affects the soil’s microbial community and population [20,22,31]. 

**Figure 2:**
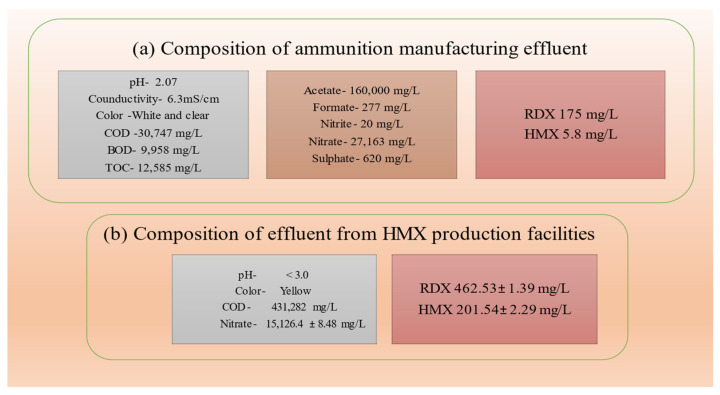
Characteristics of (**a**) ammunition manufacturing effluent [25] and (**b**) effluent of HMX production facilities [6].

Different concentrations of HMX in the soil has been reported by many researchers [14,20,33]. In the United States and Europe, HMX is recognized as a key control pollutant [26]. The US Environmental Protection Agency (USEPA) has set 0.04 mg/L as the acceptable limit for HMX in drinking water [30]. However, a significant concentration of HMX has been detected in different effluents [6,25]. To meet the guidelines and reduce the harmful effect of HMX on the environment, it is crucial to treat HMX in an eco-friendly manner. However, many obstacles are associated with the treatment of HMX effluents due to their highly acidic nature and high COD levels [6]. Wastewater from the explosive and ammunitions industries contains huge amounts of contaminants with significant concentrations of nitrate and various organic nitro-compounds such as 2,4,6- trinitrotoluene (TNT), hexahydro-1,3,5-trinitro-1,3,5-triazine (RDX), HMX, nitroglycerine, etc. In addition, various chemicals such as hexamine, CH_3_COOH, NH_4_^+^, HNO_3_, and NO_3_^−^ have been detected in the HMX production effluents. Further, RDX usually exists in HMX wastewater as a co-contaminant [6,30]. The composition of HMX-contaminated wastewater from different sites is displayed in Figure 2 [6,25]. 

Due to the heterogeneous composition of wastewater effluent of explosive production plants, it is challenging to develop an effective and inexpensive treatment technology. However, there is a growing need for the development of economically viable and efficient treatment technology for HMX-contaminated groundwater, wastewater, and soil.

## 2. HMX Remediation Approaches

In the past, several initiatives have been taken to develop a suitable approach for the treatment of HMX and other explosive chemicals in the soil and contaminated water [6,14,15,29,34]. The treatment approach can be categorized as a physicochemical or biological approach based on the treatment principle. Physiochemical remediation and bioremediation are the major approaches for HMX abatement [12,30,35]. 

### 2.1. Physicochemical Remediation

Various physicochemical remediation methods have been investigated for HMX remediation from the soil, groundwater, and wastewater. Some of them are adsorption, reduction, incineration, advanced oxidation processes (AOPs), alkaline hydrolysis, etc. [1,25,36,37,38,39,40,41,42,43,44,45,46,47]. Section 2.1 is a discussion of the various physicochemical remediation approaches; some physicochemical remediation approaches are listed in Table 2. 

#### 2.1.1. Adsorption

The adsorption technique has been extensively employed in the removal of hazardous pollutants and is acknowledged to be a very promising approach owing to its high selectivity and cost-efficacy [53,54,55,56,57]. The commercially available and synthesized activated carbon-based adsorption of toxic pollutants has been reported by many researchers [58,59]. Various low-cost materials have also been examined to develop efficient activated carbon [58]. The efficiency of granular activated carbon (GAC) for the adsorption of HMX from contaminated groundwater was evaluated using a small-scale column [36]. Different types of GACs were selected for this study and among all, the Calgon F400 sample was found to be very effective in HMX removal. Explosive-loaded GAC was regenerated by using bio-regeneration where explosive-loaded GAC was degraded in a bioreactor [59]. It was noted that HMX reduced from 0.6 mg/L to 0.4 mg/L in 4 days. Furthermore, the silica-based adsorbent was employed for HMX adsorption where ammonium perchlorate oxidation was utilized for the preparation of mesoporous silicas which showed abundant surface silanol groups [50]. The ammonium perchlorate (AP)-HNO_3_ treated SBA-15 showed 4.7 μmol/g adsorption capacity for HMX. 

In another study, HMX removal was investigated using solid-phase extraction on activated carbon prepared using spent coffee grounds [60]. Furthermore, the efficacy of various cellulosic material-based adsorbents on the adsorptive removal of various explosives, along with HMX, from stormwater was also examined [61]. Cationized pine shavings and burlap material surpassed all other materials assessed. In addition, the efficiency of commercially available cationized adsorbent materials was investigated. The efficiency of cetyltrimethylammonium chloride (CTAC) was investigated for the removal of ppb level (0.6 ppb) HMX from groundwater using a rapid small-scale column test [19]. In another study, Poly(N-isopropylacrylamide)-copoly(acrylic acid) hydrogels were synthesized for the abatement of HMX and other pollutants [52]. Batch adsorption was conducted to evaluate the removal efficiency of synthesized hydrogels. The impact of parameters, viz., temperature and pH, on the swelling of hydrogels was investigated and it was demonstrated that the hydrogels are very stable and could be a potential candidate for the removal of pollutants. 

Two different types of soils, viz., agricultural topsoil (VT, 8.4% TOC) and sandy soil (SSL, 0.33% TOC), were utilized for the study of HMX sorption and degradation [51]. HMX adsorption on soil was performed at room temperature and it was noted that the TOC content of the soil did not pose any significant impact on HMX removal. Further, the removal of HMX was investigated using different nanocomposites, viz., α-Fe_2_O_3_-rGO and nZVI-rGO [37]. Batch mode adsorption experiments were performed for HMX removal. Contact time, pH, and adsorbent dosage were among the parameters that were optimized; the Freundlich isotherm was suitable for isotherm data. 

#### 2.1.2. Reduction

Zero-valent iron is very effective in organic pollutant degradation. The ability of nanoscale Zero-valent iron (nZVI) for the treatment of HMX and other explosives from wastewater was evaluated [40]. For this study, nZVI was fabricated by the co-precipitation method. The surface area and diameter of synthesized nZVI were 42.56 m^2^/g and 20–50 nm, respectively. The LC/MS/MS technique was used for the confirmation of HMX degradation byproducts (formaldehyde/methanol/hydrazine/dimethylhydrazine). The finding of the study indicated nZVI as an excellent candidate for the in situ degradation of explosives containing wastewater. However, it is noteworthy that in the chemical reaction, corrosion of nZVIs occurs due to the formation of Fe_3_O_4_ which affects the efficiency of nZVIs. 

In another study, the role of didecyldimethylammonium bromide (didecyl) surfactant was examined for HMX destruction by zero-valent iron (Fe^0^) in contaminated soils [22]. It was revealed that a very low concentration of dodecyl (2% *w*/*v* with 3% of Fe^0^ (*w*/*v*) is required for the destruction of solid-phase HMX. Further, ZVI was utilized for HMX degradation under anoxic conditions [29]. It was demonstrated that two initial reactions, (i) reduction of the N-NO_2_ group to the five nitroso products and (ii) ring cleavage from either HMX or 1NO-HMX, could be accountable for the degradation of HMX. Finally, HCHO, NH_4_^+^, NH_2_NH_2_, and N_2_O were generated as HMX degradation byproducts. Due to the presence of NH_2_NH_2_ and HCHO, additional treatment is required. 

The reduction of HMX in soil and groundwater samples was also examined using nZVI nanocomposites [32]. Reduction experiments were conducted using different systems, viz., batch, column, and a permeable reactive barrier (PRB) system. The batch mode system offered a better performance in comparison to the other systems. HMX degradation resulted in N_2_O, CO_2_, and CH_4_ via cleavage of the ring structure. Further, a reductive technology using a bimetallic catalyst was developed for the abatement of HMX and other explosives [39]. Two-phase reactors consisting of bimetallic particles and an aqueous stream were used for the reductive technology where the bimetallic catalyst was prepared by electroless deposition. It was reported that Fe/Ni and Fe/Cu can easily degrade explosives and that the pH affects the degradation process.

#### 2.1.3. Advanced Oxidation Processes (AOPs)

The efficiency of AOP techniques for organic pollutant degradation is well documented [62,63,64,65,66]. AOPs such as photocatalysis using various kinds of photocatalysts, Fenton process, photo-Fenton, electro-assisted photo-Fenton processes, etc. have been investigated for HMX abatement [6,25,30,42,48]. 

##### Photocatalysis

TiO_2_ photocatalyst was used for HMX degradation in explosives-contaminated wastewater [43]. The influence of the initial concentration and pH on the HMX degradation and mineralization was assessed. A higher degradation of HMX was recorded in the case of low HMX concentration and a neutral pH was more effective in HMX degradation; approximately 59% TOC removal was noted for HMX. NO_3_^−^, NO_2_^−^, and NH_4_^+^ were the major byproducts. Similarly, in another report, photocatalytic degradation of HMX was conducted using UV/TiO_2_ [41]. 

Catalytic as well as advanced oxidation processes (AOPs) using UV and hydrogen peroxide were investigated for the oxidation of HMX in contaminated water [49]. Catalytic oxidation was conducted using a 4.45 wt% Pt/TiO_2_ catalyst. For UV photolysis, H_2_O_2_ was used as an oxidant and UV radiation was selected at 254 nm with experiments conducted at ambient conditions. The temperature was found to have a significant impact on both catalytic and non-catalytic oxidation. Additionally, HMX degradation by UV/TiO_2_ photocatalysis was conducted using a circular photocatalytic reactor [38]. The effect of parameters, viz., photocatalyst dose, HMX initial concentration, and initial pH, on HMX degradation was examined. It was observed that UV and TiO_2_ were not effective when used alone. A remarkable effect was achieved when TiO_2_ and UV were used together during the HMX degradation. A higher degradation rate was obtained in the neutral range of pH and degradation kinetics followed pseudo-first-order kinetics. TOC removal of 60% was obtained within 150 min and higher degradation was observed when the TiO_2_ dose was 0.7 g/L. With a 5 mg/L concentration of HMX, the recovery of nitrogen was approximately 70%. Analysis of the degradation byproducts confirmed the presence of NO_3_^−^, NO_2_^−^, and NH_4_^+^ in the treated samples.

##### Fenton and Photo-Fenton

**Figure 3:**
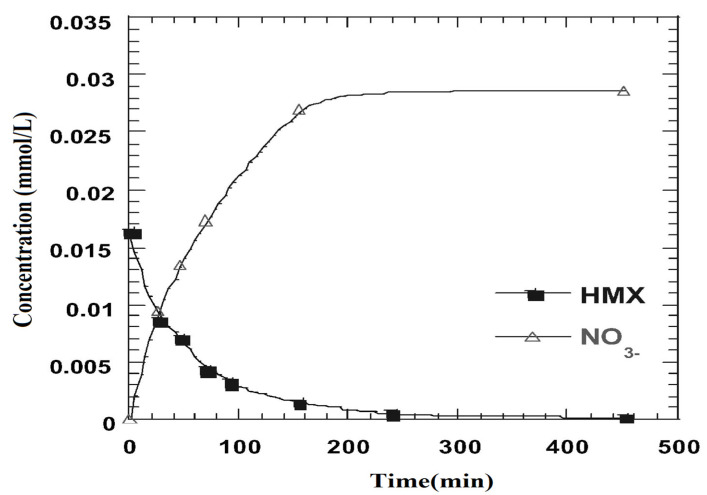
HMX degradation kinetics by Fenton oxidation. Adapted from Ref. [30] with permission from Elsevier.

The Fenton and photo-Fenton processes are effective in the abatement of numerous organic pollutants, including explosives residue in water bodies and soil [42,67,68]. In the study of Bhanot et al., the efficiency of different AOPs, viz., photolysis, photo-peroxidation, and photo-Fenton oxidation, for the degradation of HMX from wastewater were examined [6]. The concentration of nitrate and chemical oxygen demand (COD) was determined in treated samples. Both photo-peroxidation and photo-Fenton oxidation treatment approaches were able to remove approximately 98% of HMX from wastewater [6]. Zoh and Stenstorm investigated the Fenton oxidation of HMX at pH 3.0 [30]; the resulting kinetic data fit well with the pseudo-first-order equation (Figure 3). 

It was reported that the concentration of Fenton’s reagent affects the reaction rate. The findings of this report demonstrated that Fenton’s reagents can efficiently mineralize HMX, resulting in the formation of nitrate as a byproduct of HMX oxidation. It was reported that 1.75 M NO_3_^−^ was generated per M HMX in the Fenton process when the experimental parameters were as follows [Fe^2+^] 0.72 mmol/L, [H_2_O_2_] 77.6 mmol/L, and HMX molar concentration 0.015 mmol/L. Similarly, in another investigation, HMX-containing wastewater was treated by the Fenton process [48]. Effects of H_2_O_2_ dosage, FeSO_4_·7H_2_O dosage, reaction time, and pH were examined and optimized for maximum degradation. A temperature of 25 °C; pH 3.0, 0.2 mL of 1% H_2_O_2_; 8.3 mL of 0.01% FeSO_4_·7H_2_O concentration, and a reaction time of 80 min were found to be optimum for the degradation of a 4 mg/L HMX concentration. The efficiency of Fenton and photo-Fenton processes for HMX and other explosive degradation was evaluated by Liou et al. [42]; enhanced HMX degradation was achieved when a higher concentration of Fe (II) was used. 

**Figure 4:**
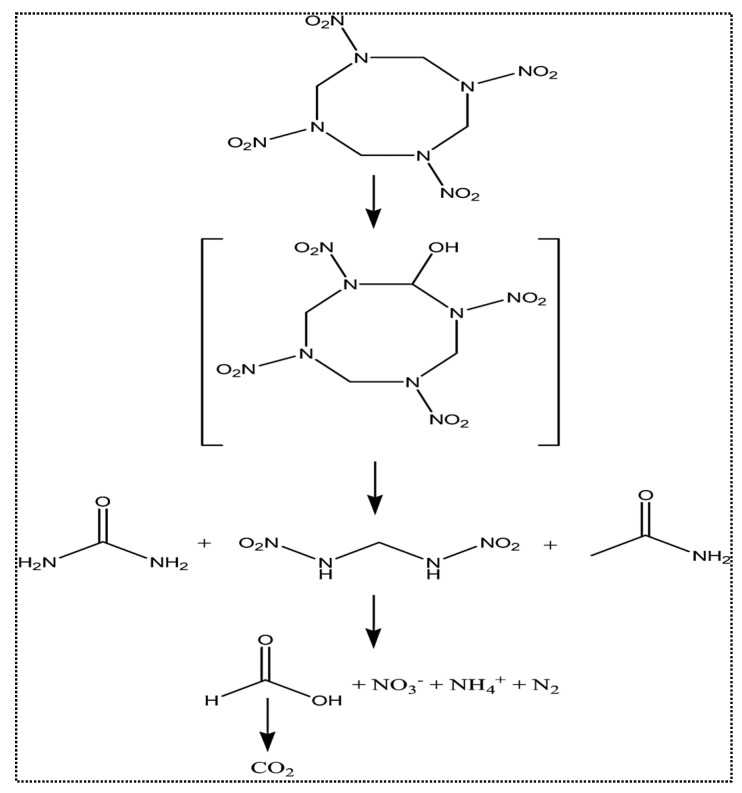
The HMX degradation mechanism in electro-assisted Fenton treatment. Adapted from Ref. [25] with permission from Elsevier.

Furthermore, the electro-assisted Fenton process was utilized for HMX degradation from actual wastewater [25]. The electro-assisted Fenton process resulted in a higher degradation in comparison to Fenton and photo-Fenton processes. In the electro-assisted Fenton process, hydroxyl(^•^OH) radicals generate on the anode surface. It was stated that the biodegradability of effluents was enhanced after Fenton treatments which makes the further treatment of effluent by a biological approach more feasible. Formic acid and three amino derivatives were identified as intermediate byproducts and after complete mineralization, NO_3_^−^, NH_4_^+^ CO_2_, and water were detected in treated samples. The HMX degradation mechanism is presented in Figure 4 [25]. In HMX degradation, at the initial step, the carbon atoms of the heterocyclic ring are hydroxylated and the opening of the heterocyclic nitramines ring results in the generation of formic acid, methylene dinitramine, urea, and acetamide. 

#### 2.1.4. Other Physiochemical Approaches

Several other approaches such as incineration, alkaline hydrolysis, and utilization of subcritical water for HMX degradation have been explored by many researchers [1,20,69,70]. The incineration technique has been widely used in past for the abatement of explosive-contaminated soil, but this approach is affiliated with NOx emissions which is the main downside of this technique [69,71]. Recently, rotary kiln incineration has been reported, which demands less space than conventional incineration where hot gas is used to break down explosive waste. However, similar challenges such as NOx emissions remain intact. For the treatment of explosive waste, fluidized beds perform better than rotary kilns [69,70]. 

Alkaline hydrolysis is an economically feasible approach for HMX-contaminated water. The HMX degradation mechanism in alkaline hydrolysis was examined in [1]. In this study, HMX was hydrolyzed in an aqueous solution (pH 10–12.3) and the information on the generated byproducts and degradation pathways was presented. In another study, 57% elimination of HMX was reported for alkaline hydrolysis [21]. In addition, Heilmann et al. investigated the alkaline hydrolysis of HMX [72]. For HMX hydrolysis kinetics, the temperature varied from 50 to 80 °C while the pH range was selected in the alkaline range from 10–12. 

A pilot-scale study for the treatment of different explosives in soil was conducted using subcritical (hot/liquid) water [20]. For this study, soil samples were gathered from defense sites. Significant degradation of HMX was obtained at 125 °C using subcritical water. It was reported that HMX degradation generates byproducts that eventually degraded to a 99.9% destruction and ca. 98% destruction of HMX. A toxicity assessment test of processed wastewater or soil leachates using *Vibrio fischeri* in Microtox” acute toxicity tests demonstrated no sign of toxicity. 

### 2.2. Bioremediation

Another widely employed remediation approach for HMX treatment in soil, groundwater, and wastewater is bioremediation, which includes aerobic/anaerobic biological treatment, phytoremediation, soil mud reactor treatment and composting, etc. [8,34,73,74,75,76,77,78]. Biological treatment has been recommended as a potential approach for the treatment of HMX contaminants as an environmentally sustainable alternative [23,79,80]. In this section, we have reviewed microbial remediation and phytoremediation approaches. 

Two bioremediation methods, viz., soil slurry reactor and land farming technique were assessed for explosives-contaminated soil remediation [23]. The selected soil was contaminated with varied explosives (TNT, RDX, and HMX) and the concentration of HMX in the contaminated soil was approximately 900 mg/kg of pH 6.5 soil. Although the chosen methodology was effective in reducing HMX concentrations in soil, the removal efficiency was slightly lower in contrast to other explosives (TNT and RDX). HMX biodegradation has been explored across both aerobic and anaerobic conditions in several studies [4,9]. In numerous research investigations, various microbial species have been explored for HMX remediation (Table 3).

The HMX degradation in aerobic conditions was investigated by using *Planomicrobium flavidum* strain S5-TSA-19, which was extracted from explosive-contaminated soil [4]. Approximately 70% of HMX was degraded in 20 days and methylenedintramine (M.wt.−136 Da) and N-methyl-N,N′-dinitromethanediamine (M.wt.−149 Da) were produced from HMX degradation. It was reported that the first-order kinetics fitted well with the HMX degradation kinetic data. Furthermore, HMX aerobic biodegradation from groundwater was studied. It was demonstrated that microbial consortia decreased the HMX concentration from 6 to 1 mg/L within 5.2 days [84]; five metabolites were identified from HMX biodegradation. 

In another study, *Phanerochaete chrysosporium* was used for the aerobic biodegradability of HMX [83]. It was reported that 600 nmol of HMX can be degraded in 25 days of incubation time when the 7 days old static *P. chrysosporium* liquid cultures were used for biodegradation [83]. HMX reduction was proposed via the formation of its mono-nitroso derivative (1-NO-HMX). Two possible routes for HMX degradation were suggested. The first possible route involved N-denitration followed by hydrolytic ring cleavage, while R-hydroxylation followed by ring cleavage was reported as the second route. 4-nitro-2,4-diazabutanal (NDAB), HCHO, NO_2_^−^, and N_2_O, were obtained from 1-NO-HMX degradation. Two different soil samples contaminated with HMX were tested and considerable mineralization was achieved in the presence of fungus. The present findings demonstrate the utility of the fungus *P. chrysosporium* for HMX remediation. 

Wastewater generated from HMX production was treated using the aerobic granules in an aerobic granular reactor and it was demonstrated that aerobic granular sludge was effective in the separation of organic matter (97.57%) and nitrogen compounds (80%) within 40 days of treatment time [18]. In contrast to aerobic conditions, anaerobic treatment degrades HMX more effectively [15]. HMX degradation was investigated using mesophilic anaerobic granules [5]. Biotic and abiotic mechanisms were both attributed to the HMX degradation under mesophilic conditions. It is reported that 99.04% removal was achieved using volatile suspended solids/L acclimated while 96.42% removal was obtained when unacclimated granules were used. A considerable contribution of adsorption was observed in the abiotic process. An inhibitor of methanogenic bacteria (2-bromoethanesulfonic acid) affected the biotransformation of HMX and a slight inhibition in metabolic activity was reported. A significant impact was noted in the presence of an inhibitor of acetogenic bacteria (*Vancomycin*). HMX degradation in the presence of nitrate and sulfate was also investigated and it was observed that nitrate had a significant effect on HMX biotransformation by anaerobic granules while sulfate had a minimal effect. 

In another study, the impact of carbon substrate, viz., CH_3_COOH, C_2_H_5_OH, C_6_H_12_O_6_, and soluble starch on HMX biodegradation was explored [27]. A batch system was used for the biodegradation of HMX using anaerobic mesophilic granular sludge. Glucose and acetate sources offered a better performance in contrast to ethanol and soluble starch. The carbon source concentration was found to be very critical in the biodegradation of HMX. Further, Liu et al. explored the effect of various co-substrates on the degradation of HMX from simulated wastewater under anaerobic conditions [74]. Enhanced biodegradation was observed in the existence of a dextrose and acetate co-substrate. Using a dextrose co-substrate, 98.5% degradation of HMX was accomplished in 7 days of treatment time while decreased HMX degradation was recorded when sodium nitrate was used as the co-substrate.

The effectiveness of *Bacillus aryabhattai* in the biotransformation of HMX was studied in [26]. The tolerance of *Bacillus aryabhattai* against HMX was found to be very high. It was demonstrated that HMX biotransformation takes place outside the bacterial cell. However, in presence of HMX, there was a metabolic imbalance in cells for lipids and lipid-like molecules. *Bacillus aryabhattai* inoculation led to a 90.5% removal of HMX within 24 h for 5 mg L^−1^ of HMX concentration. FTIR analysis showed the presence of the –OH functional group on the bacterial cell surface. HMX biotransformation in the presence of an enzyme was also explored [9]. Xanthine oxidase was utilized as a catalyst for HMX biotransformation, and it was observed that anaerobic conditions were more effective in comparison to aerobic conditions, and the biotransformation rate in anaerobic conditions was recorded to be 10.5 ± 0.9 nmol h^−1^ mg protein^−1^.

Furthermore, the biodegradation of HMX was investigated in different electron-acceptor conditions [7]. The sewage treatment plant’s based anaerobic digester was utilized for the development of culture. HMX biodegradation was tested under different conditions, viz., mixed electron-accepting conditions, methanogenic, fermenting, sulfate-reducing, and nitrate-reducing conditions. Methanol and chloroform were the end-products of HMX biodegradation when the mixed electron-acceptor conditions were used. A reductive pathway for HMX treatment using organic mulch (microorganism consortium) as an electron donor was also investigated [17]. In the column study with 8 ppb influent HMX, complete removal was recorded. Mulch and compost introduced to aquifers can establish an anaerobic environment and act as an electron donor, supporting the conversion of electrophilic contaminants via reductive pathways. Mulch is a low-cost naturally available electron donor which surpassed other viable options. When HMX-contaminated wastewater flows via in situ mulch PRB, it works like a slow-release source for soluble carbon electron donors which improves the HMX biodegradation.

Bioaugmentation is an effective approach for in situ remediations of contaminated soil. Eco-friendly carriers for the immobilization of microbes have a significant impact as they protect the microbes from undesirable pH conditions and the presence of hazardous compounds. The bioaugmentation approach was tested for the abatement of HMX-contaminated soil [8]. For this study, a mixture of coca peat and calcite was used for the immobilization of the soil bacterium “*Janibacter cremeus*”. Under aerobic and anoxic conditions, HMX was degraded in 35 days. Mass spectrometric (MS) analysis confirmed the presence of nitroso derivatives from the anoxic degradation of HMX. Two different pathways, viz., (a) two-electron reduction pathways [51] and (b) denitraion pathway [1], were proposed for HMX degradation. 

The potential of the bacterial strain, *Bacillus toyonensis* on the HMX contaminated site was tested for HMX degradation in an aqueous medium [31]. Response surface methodology was chosen for the experimental design in this study. Parameters, viz., microbial inoculum size, degradation time, and HMX initial concentrations, were optimized. For 2 mg/L HMX, 87.7% degradation was recorded in 15 days. The determination of nitrite and nitrate concentrations confirms the HMX degradation. Anaerobic oligotrophic conditions for the biodegradation of HMX in cold marine sediment were also evaluated [85]. For a 1.2 mg/L HMX concentration, it took 50 days for a 50% reduction in HMX concentration. Improved HMX removal was noted with the availability of glucose as a carbon source. The potential of soil bacteria available in the contaminated site was used for the HMX removal from soil samples [15]. In presence of molasses as a co-substrate, HMX was degraded by soil bacteria via a co-metabolic process. A batch mode study showed that a 97% reduction in HMX concentration was achieved in 4 months of reaction time.

The biological nitrification of alkaline hydrosylate of HMX and RDX was also examined [86]. A denitrifying (anoxic) packed bed upflow reactor was used for the alkaline hydrosylate treatment of HMX which generated acetate, formate, formaldehyde, and nitrite. Within 3 h of retention time, 90% removal of organic compounds and nitrite from hydrosylate was ascertained. Denitrifying bacterial isolates of Pseudomonas (HPB1) and Bacillus (HPB2 and HPB3) were tested for HMX biodegradation [77]. The effluent for this study was collected from an HMX production plant. The wastewater characterization showed the presence of CH_3_COOH, NH_4_NO_3_, explosive residue, and other organic nitro bodies. HMX was efficiently degraded by the isolate HPB1. The HPB2 performed admirably in HMX-containing effluent when neutralized with NaHCO_3,_ while samples neutralized with ammonia were not suitable for biotransformation and resulted in a lower degradation of HMX. However, denitrifying HPB1 was effective in the nitrate reduction for both the neutralizing agents. 

The treatment of explosives-contaminated aquifer slurries was carried out using anaerobic biodegradation. For this purpose, C_2_H_5_OH and propylene glycol were selected as electron donors which ultimately support providing syntrophically produced H_2_ which participates in the HMX degradation anaerobically [87]. The slurries of explosives contaminated groundwater and soil and were used for the construction of anoxic microcosms. Enhanced biodegradation of HMX and other explosives was recorded when 5 mM C_2_H_5_OH and propylene glycol were used. C_2_H_5_OH depletion produces H_2_, CH_3_COOH, and CH_4_ in 20 days while propylene glycol produces H_2,_ CH_3_COOH, and propionate as end-products after 15 days of degradation time. The presence of ethanol and propylene glycol in slurries provides H_2_ which enhances the biodegradation of HMX in the explosive-contaminated soil.

Further, for anaerobic HMX degradation, the performance of ruminal microorganisms from whole rumen fluid (WRF), and 23 commercially available ruminal strains was examined [88]. It was observed that in 24 h of degradation time, the concentration of HMX was reduced from 30 µM to 5 µM HMX. In HMX degradation, firstly nitroso or hydroxylamino intermediates were formed by HMX reduction followed by enzymatic ring cleavage to other byproducts. The HMX degradation ability to metabolize the bacteria of two unexploded ordnance (UXO) disposal sites (UXO-3 and UXO-5) and one reference site (midref) sediment was investigated [73]. Two different groups of bacteria (group I- Aerotolerant anaerobes and microaerophiles and group II facultative anaerobes) were confirmed in the UXO-5 sample by 16S rRNA sequencing. Anaerobic bacteria (group III and group IV) were available in UXO-3 and midref sediments samples. Bacteria species in different groups were as follows: group I- phylogenetic cluster of Clostridiales; group II- Paenibacillus; group III- Tepidibacter of Firmicutes; and group IV- Desulfovibrio of Deltaproteobacteria. It was observed that approximately 26.8% of HMX was mineralized by group IV bacteria in 308 days while other bacteria from other groups gave negligible mineralization. 

#### Phytoremediation 

Phytoremediation is environmentally friendly, economically viable, and a feasible alternative for the removal of explosives and is an acknowledged method used for environmental remediation [12,24,89]. The phytoremediation approach includes the utilization of green plants for the remediation of water and soil. Plant enzymes play a vital role in the oxidation/reduction of pollutants [89,90]. The uptake and biotransformation of pollutants in phytoremediation can be differentiated as phytoextraction, phytovolatilization, phytodegradation, rhizofiltration, and phytostabilization [91,92]. Usually, explosives are environmentally stable and resistant to remediation. HMX is less vulnerable to plant uptake in comparison to other explosives [28]. Various factors such as HMX concentration, ionization constant, pH of sample solutions, organic matter content, and plant physiology affect the phytoremediation of HMX [12]. A long remediation process time makes phytoremediation a time-consuming approach [12,91]. 

HMX uptake by hybrid poplar trees was demonstrated and various parameters for HMX uptake were investigated [11]. The HMX uptake in hybrid poplar trees was achieved through hydroponic solutions. It was observed that HMX uptake did not show any toxic effect on hybrid poplar cuttings. For HMX uptake confirmation, radiolabeled [U-14C] HMX was utilized. Further, in another study, *Myriophyllum aquaticum* and axenic hairy root cultures of *Catharanthus roseus* were used for HMX uptake [93]. The exposure level for HMX was set as 5 mg/L. Additionally, *Methylobacterium* sp. (strain BJ001) extracted from hybrid poplar tissues (Populus deltoides X nigra DN34) was applied for HMX degradation and it was reported that the *Methylobacterium* sp. was efficient in the transformation of 2.5 mg/L of [U-14C]HMX within 40 days [35]. Further, soil from an anti-tank firing range was utilized for growing indigenous terrestrial and some agricultural plants which were then utilized for the HMX degradation [28].

The potential of alfalfa (*Medicago sativa*) for explosive biodegradation was investigated and it was reported that HMX can be degraded by hydrolase secreted by alfalfa [75]. The degradation rate for HMX by alfalfa was 18.4% under hydroponic conditions. It has been reported that Methylobacterium populum sp. nov., strain BJ001 can degrade HMX and other explosives compounds efficiently [76]. Because autotroph plants lack enzymatic mechanisms to effectively metabolize organic contaminants, the phytoremediation approach is typically slow and incomplete [12]. In genetically modified plant species (transgenic plants), the potential of bacterial genes to degrade organic pollutants is integrated with the plants’ phytoremediation advantages. In recent years, the biodegradation of explosives using transgenic plants has sparked great attention due to their higher efficiency and tolerance towards explosives [89]. The introduction of bacterial genes, for example, nitroreductase and cytochrome P450, into plants can enhance the tolerance level of plants and degradation performance [12]. Phytoremediation could be a promising approach in the treatment of explosive-contaminated wastewater/soil and more exploration is needed for further development in transgenic plants.

## 3. Byproducts/End-Products of HMX Degradation Via Different Treatment Approaches 

It is clear from the discussion of physicochemical and bioremediation approaches that the degradation pathway is highly dependent on the treatment approach and process parameters [94,95,96,97]. The intermediate/end-products of HMX degradation in various remediation methods are shown in Table 4. It is clear from this table that NO_3_^−^, NO_2_^−^, and NH_4_^+^ are found in the majority of the investigations, raising the possibility that secondary treatment may be required to avoid releasing these species into the environment.

## 4. Advantages and Disadvantages of HMX Remediation Approaches

Owing to their ever-increasing demand and a continual increase in production, HMX and other energetic materials have led to environmental pollution [3,73,75]. Due to environmental legislation and the toxicity of these contaminants, there is a growing need for the development of a sustainable approach for their removal from contaminated soil and wastewater/groundwater [6,30]. HMX has been effectively removed from the contaminated soil and wastewater by various approaches. However, these approaches have some advantages as well as disadvantages that need to be taken into consideration during the selection of any treatment tool for the remediation of HMX-contaminated soil or wastewater (Table 5)**.**

The advantages of physicochemical methods include their fast treatment speed and wide applicability on the wider range of HMX concentrations in soil/groundwater/wastewater, and, so far, they have become the preferable methods for the remediation of HMX. Incineration is a very effective treatment approach, but it is associated with high energy consumption. Additionally, incineration can cause NO*_x_* emissions and affect soil fertility. Furthermore, incineration is an expensive treatment method for HMX abatement [20]. Adsorption is an effective approach for HMX removal; however, it results in the separation of pollutants rather than their destruction. Chemical reduction and advanced oxidation processes (AOPs) are effective in terms of degradation, but during treatment, the generation of toxic byproducts usually requires secondary treatment [102]. Some conventional treatment approaches are energy-intensive and not economically viable and are associated with the generation of toxic end-products [2,27,30]. 

The biological method for the removal of hazardous pollutants such as explosive wastes has been increasing in demand resulting in the exploitation of the potential of various microorganisms and plant species [74]. Owing to the low-cost and environmentally friendly processes, the biological treatment approach is very promising for the remediation of HMX-contaminated soil and water [26]. In the biological approach, microorganisms utilize explosives as their nitrogen source, but it is time-consuming [74]. In some cases, microbial consortiums are not very tolerant to explosives and can be inactivated by toxic pollutants.

Phytoremediation is another environmentally friendly approach and has many advantages over other technologies [74]. Explosives can be mineralized using phytoremediation. It was reported that the cost for phytoremediation of explosive contaminants is half that compared to any other treatment approach. The cost for soil remediation using phytoremediation was estimated at $25–$100/ton while the cost estimated for the treatment of wastewater has been reported as $0.60–$6.00/1000 gallons of wastewater [91]. However, phytoremediation requires a longer time for growing plants [26]. As plant species and plant growth are very much dependent on the climate and geological conditions, it is very much limited for a particular site. Further, HMX and byproducts of HMX may accumulate in the plant and may thus persist in the environment. 

## 5. Challenges

The role of Octahydro-1, 3, 5, 7-tetranitro-1, 3, 5, 7-tetrazocine (HMX) in different fields is well recognized. Due to the extensive applications of HMX, different concentrations of HMX are found in soil, groundwater, and wastewater. Because of its recalcitrant nature, HMX can sustain itself in the environment for a long period. Diverse approaches to HMX mineralization, such as AOPs or bioremediation, typically produce nitrate, nitrite, and ammonium. There is a serious environmental issue associated with these end-products which demand removal, typically via biological treatment processes. Such combinations of processes should be studied together. On the other hand, after AOPs, there is a risk of the formation of disparate nitro-products due to reactive nitrogen species, and the possibility of a reaction of nitrogen species with other byproducts or residual parent products; thus, undesired toxic end-products can be formed. Therefore, meticulous monitoring of these end-products is critical, and appropriate abatement should be considered for secondary treatment to avoid the discharge of nitro-products. The degradation pathway and intermediates of explosive contaminants need to be thoroughly understood. In terms of AOPs, several very effective approaches were still not applied for HMX degradation. This relates to cavitation-based AOPs which proved synergism in the case of the removal of many groups of organic compounds [103]. A comparable research gap exists concerning sulfate radical-based AOPs (SR-AOPs) which have already been proven to be effective and sustainable oxidants [96,97]. On the other hand, HMX bioremediation approaches such as aerobic/anaerobic degradation and phytoremediation are environmentally friendly, but these treatments require a long time. Additionally, microbial species/plant species are sensitive and need careful assessment.

## 6. Conclusions 

The application of HMX in different fields is unavoidable, hence its proper treatment is the only solution for a safe environment. There is a growing concern about developing new, cutting-edge, and sophisticated technologies for HMX remediation. Technologies such as advanced oxidation processes (AOPs), reduction, incineration, adsorption, etc. have been investigated by many scientific researchers and found to be effective in HMX treatment. However, most of the technologies are associated with a high-cost factor and additional environmental problems. Further, the development of sustainable materials for HMX abatement is complicated. Owing to the pH-dependent surface charge of photo-catalysts/adsorbents, the selection of efficient photo-catalysts/adsorbents is challenging.

Although sulfate radical-based advanced oxidation processes have turned out to be remarkably efficient in the mineralization of a variety of organic pollutants, their appropriateness for HMX treatment has not been adequately scrutinized. Bioremediation for HMX treatment has gained momentum in the past few years; however, the uptake mechanism and effect of reaction intermediates/byproducts on microbial species/plant species still need better understanding. In recent years, researchers have explored the possibilities of transgenic plants for the abatement of HMX which can enhance the treatment time and mineralization issues. However, there are many hurdles related to the transgenic plant-based remediation approach that needs to be explored. Finally, from an industrial point of view, more initiatives are required to explore simplified, reliable, and cost-effective strategies for HMX abatement in a safer manner. The integration of different techniques in a series as a pre-or post-treatment can facilitate the high removal and full mineralization of HMX. Despite the remarkable developments in recent years, some obstacles remaining in HMX treatment technologies must be resolved to make the HMX treatment approach safer, more effective, and sustainable.

## Figures and Tables

**Table 1:** The concentration of HMX in groundwater, wastewater, and soil samples.

Samples	Source	HMX Concentration	References
Groundwater	HMX-contaminated groundwater sample from the Pueblo Chemical Depot (PCD), Colorado	9.03 µg/L	[17]
Groundwater	Sample from a well in eastern Massachusetts	0.6 ppb	[19]
Soil	Soil sample from a defense site	0.08 wt% of HMX	[20]
Soil	HMX-contaminated soil from the Iowa army ammunition plant	700 mg/kg	[15]
Soil	Soil samples from munitions plants and firing ranges (Nebraska Ordnance Plant)	6.36 ± 1.71 mg/kg	[21]
Soil	Soil samples from a high-explosive (HE) manufacturing and testing site	45,000 mg/kg	[22]
Soil	Soil samples from the LAAP in Minden, USA	600 to 900 mg/kg	[23]
Wastewater	Munition facility wastewater effluents	12 ppm	[24]
Wastewater	Wastewater sample from an HMX production plant	8.23 mg/L	[18]
Wastewater	Ammunition manufacturing effluent	5.8 mg/L	[25]

**Table 2:** Physicochemical remediation approaches for HMX abatement.

HMX Remediation Approach	Conditions	Medium	Removal/Degradation Efficiency/Adsorption Capacity	Reference
Fenton process	Temp. 25 °C; 0.2 mL of 1% H_2_O_2_; 8.3 mL of 0.01% FeSO_4._7H_2_O, pH 3.0; reaction time 80 min; HMX concentration 4 mg/L, and COD 214 mg/L	Wastewater	81.4%	[48]
Zero-valent iron	4% Fe^0^ (*w*/*w*) + 2% didecyl (*w*/*v*) cationic surfactant, HMX 45,000 mg/kg, and reaction time 6 days	Soil	>80%	[22]
UV and hydrogen peroxide	4.45 wt% Pt/TiO_2_ catalyst, particle diameter < 105 μm, surface area of 62.8 m^2^/g batch test; UV radiation 254 nm, stirring 300 rpm, temp 32 °C, pH 3.4–10.4, and HMX 4 ppm	Wastewater	NA	[49]
Electro-assisted Fenton	Ti/RuO_2_-IrO_2_ anode, HMX 5.8 mg/L, and electroactive area 37 cm^2^	Wastewater	60%	[25]
Fe/Cu bimetal reduction	Volume 60 mL, 600 mg bimetallic particles; 1% solid/liquid loading, pH 3.0, and HMX conc. 4.98 mg/L	Synthetic solution	NA	[39]
Adsorption- mesoporous silicas	HMX conc. 21 mg/L, 0.1 g N-SBA-15, contact time 30 min, temp 20 °C, and agitation speed 250 rpm	Aqueous solution of explosives	4.7 μmol/g adsorption capacity	[50]
Fenton process	Temp 20 °C and 50 °C, pH 3, HMX conc. 4.5 mg/L, and molar ratios of 5178: 48:1 of Fe^2+^: H_2_O_2_: HMX	HMX solution		[30]
Granular activated carbon (GAC)	350 μg/L HMX and small-scale column tests	Groundwater	NA	[36]
Photo-Fenton process	H_2_O_2_ to FeSO_4_ and 7H_2_O ratio (1:1, 1:3 and 3:1); UV irradiation: 125 W and HMX conc. 201.52 ± 2.29 mg/L	Real wastewater	98%	[6]
Adsorption on soil	2 g of dried soil, HMX conc. 0.5 to 4 mg/L, and contact time 1 h to 7 d.	Synthetic solution	96%	[51]
Fenton and photo-Fenton processes	H_2_O_2_ concentration of 0.29 M and Fe^2+^ conc. 0.72 mM, temp (25 + 2 °C), speed 130 rpm, UV wavelength 254 nm, pH 2.8, and HMX conc. 1.07 × 10^−4^ M	Synthetic solution	84.9%	[42]
Poly(N-isopropylacrylamide)-copoly(acrylic acid) hydrogels	HMX conc. 5.3 mg/L, hydrogel mass 0.0024 g, volume of HMX solution 5 mL, and batch experiments	Synthetic solution	48%	[52].

**Table 3:** Bioremediation approaches for HMX removal.

Microbial Species	Conditions	Biodegradation/ Mineralization Efficiency	Reference
*Bacillus* *aryabhattai*	HMX conc. 5 mg/L and inoculation time 24 h	90.5%	[26]
*Clostridium* sp. strain EDB2	HMX 20 μM, 5 mg wet biomass ml^−1^, incubation temperature 30 °C, and chemotaxis-mediated biodegradation	8% mineralization	[81]
*Pelomonas aquatica* strain WS2-R2A-65	Incubation period 20 days, HMX conc. 6 mg/L, aerobic condition, and co-metabolism	78%	[82]
*Janibacter cremeus*	HMX conc. in a spiked sample of soil 3000 mg/kg, incubated temperature 35 °C, and incubation time 35 days	40%	[8]
*Planomicrobium flavidum* strain S5-TSA-19	HMX conc. 6 mg/L, incubated temperature 35 °C, and agitation speed 120 rpm for orbital shaker	70%	[4]
*Pseudomonas* *(HPB1) and Bacilllus (HPB2 and HPB3)*	Incubation time 60 days, incubation temperature 30 ± 2 °C, SB-HMX 0.91 mg/L (HMX effluent sample neutralized with sodium bicarbonate), and AM-HMX 0.59 mg/L (HMX effluent sample neutralized with ammonia)	For HPB1: 76.3% (SB-HMX) 27.7%(AM-HMX) For HPB2 62.9%(SB-HMX)	[77]
*Phanerochaete chrysosporium*	HMX conc. 600 nmol, incubation time 25 days, and HMX conc. in real soil samples (HMX-403 µmol/kg) and (HMX-3057 µmol/kg)	97% 75% 19.8%	[83]

**Table 4:** Byproducts/end-products of HMX degradation via different treatment approaches.

Treatment Approach	HMX Degradation Byproducts (Intermediate/End-Products)	References
Biodegradation of HMX by *Planomicrobium flavidum*	NO_2_^−^, methylenedintramine, and N-methyl-N,N′-dinitromethanediamine	[4]
Alkaline hydrolysis	NO_2_^−^, N_2_O, NH_3_, N_2_, and HCOOH	[1]
Bioaugmentation using *Janibacter cremeus,* an immobilized mixture of calcite and cocopeat for bioaugmentation.	Nitroso derivatives (5-hydroxy-4-nitro-2,4-diazapentanal and NDAB (further breaks down to HCHO)	[8]
Biodegradation by sediment microorganisms	Mononitroso derivatives	[85]
Degradation by TiO_2_ photocatalysis	NO_3_^−^, NO_2_^−^, and NH_4_^+^	[38]
Reduction by nZVI	Formaldehyde/methanol/hydrazine/dimethyl hydrazine	[40]
Electro-assisted Fenton treatment of HMX	HCOOH, NO_3_^−^, NH_4_^+^, andCO_2_	[25]
Biodegradation under the mixed electron-acceptor condition	Under mixed electron-acceptor conditions, the major metabolites were CHCl_3_ and CH_3_OH. Under methanogenic, fermenting, sulfate, and nitrate-reducing conditions, mono-, di-, and tri-nitroso derivatives were produced from HMX	[7]
Fenton oxidation	NO_3_^−^ and N_2_	[30]
Xanthine oxidase catalyzed biotransformation	NO_2_^−^, methylenedinitramine (MDNA), 4-nitro-2,4-diazabutanal (NDAB), HCHO, N_2_O, HCOOH), and NH_4_^+^	[9]
Nitrite and nitrate	NO_2_^−^ and NO_3_^−^	[31]
Photocatalytic degradation	NO_2_^−^, NO_3_^−^, and NH_4_^+^	[43]
Reduction by zero-valent Iron	HCHO, NH_4_^+^, N_2_O, and NH_2_NH_2_	[29]

**Table 5:** Advantages and disadvantages of HMX treatment approaches.

Method	Advantages	Disadvantages	References
Adsorption	Simple, low-cost, possibility to tailor adsorbent characteristics, fast kinetics, and effective in the removal of a wide range of concentrations.	Use of chemicals in the regeneration of adsorbent, treatment of exhausted adsorbent, and secondary treatment of regenerated solution consisting of explosive residues/concentrations.	[19,50,98]
Advanced oxidation processes (AOPs)	Efficient, in situ production of radicals, UV light in photo-Fenton can enhance degradation, efficient mineralization of the pollutants, Fenton/photo-Fenton-based treatment is effective, and possibility of tailoring the catalyst according to pollutant species to enhance the catalytic activity.	Technical constraints, formation of byproducts, need for secondary treatment, catalyst cost can increase overall cost, chemicals required in Fenton and photo-Fenton process, generation of iron sludge, pH-sensitive, a UV lamp can add more cost, an interfering component can affect efficiency, recovery of catalyst, formation of byproduct, and catalyst corrosion.	[6,41,98,99,100,101]
Incineration	Simple, fast, effective, and useful for concentrated effluents.	Initial investment cost, expensive, energy-intensive, secondary pollution such as harmful emissions, and possible effect on soil fertility.	[30,98]
Aerobic/ anaerobic biodegradation	Effective, low-cost, economically attractive, and well-accepted by the public.	Time-consuming, mineralization issue, degradation products, the complexity of the microbiological mechanism, generation of biological sludge, and sensitivity to pH, temperature, and concentration.	[30]
Chemical oxidation	Effective, effective mineralization, a variety of catalysts available and can be tailored according to need, and the mechanism via chemical oxidation is well explored.	Catalyst corrosion, toxic byproduct, secondary treatment needed for byproduct removal, cost of catalyst, and management of used catalyst.	[98]
Bioaugmentation	Enhanced remediation by using genetically engineered microbes.	Survival of microorganisms in different environments and delivery of the microorganism into the desired location.	[8]
Phytoremediation	Easy assessment through simple morphological visualization or by collection and analysis of cells/tissue.	Time-consuming and accumulation of explosives in plant species.	[89]

## Data Availability

Not applicable.
